# Nursing knowledge of essential maternal and newborn care in a high‐mortality urban African setting: A cross‐sectional study

**DOI:** 10.1111/jocn.14695

**Published:** 2018-11-26

**Authors:** Georgina A. V. Murphy, David Gathara, Ann Mwaniki, Grace Nabea, Jacintah Mwachiro, Nancy Abuya, Mike English

**Affiliations:** ^1^ Centre for Tropical Medicine and Global Health Nuffield Department of Medicine University of Oxford Oxford UK; ^2^ Kenya Medical Research Institute/Wellcome Trust Research Programme Nairobi Kenya; ^3^ Nairobi City County Government Nairobi Kenya

**Keywords:** Africa, emergency obstetric and neonatal care, health facilities, Kenya, maternal care, midwifery, neonatal care, neonatal nursing, Newborn care, nursing knowledge

## Abstract

**Aims:**

To assess the knowledge of nurses of national guidelines for emergency maternity, routine newborn and small and sick newborn care in Nairobi County, Kenya.

**Background:**

The vast majority of women deliver in a health facility in Nairobi. Yet, maternal and neonatal mortality remain high. Ensuring competency of health workers, in providing essential maternal and newborn interventions in health facilities will be key if further progress is to be made in reducing maternal and neonatal mortality in low‐resource settings.

**Design:**

Cross‐sectional survey.

**Methods:**

Questionnaires comprised of clinical vignettes and direct questions and were administered in 2015–2016 to nurses (*n* = 125 in 31 facilities) on duty in maternity and newborn units in public and private facilities providing 24/7 inpatient neonatal services. Composite knowledge scores were calculated and presented as weighted means. Associations were explored using regression. STROBE guidelines were followed.

**Results:**

Nurses scored best for knowledge on active management of the mother after birth and immediate routine newborn care. Performance was worst for questions on infant resuscitation, checking signs and symptoms of sick newborns, and managing hypertension in pregnancy. Overall knowledge of care for sick newborns was particularly low (score 0.62 of 1). Across all areas assessed, nurses who had received training since qualifying performed better than those who had not. Poorly resourced and low case‐load facilities had lower average knowledge scores compared with better‐resourced and busier facilities.

**Conclusion:**

Overall, we estimate that 31% of maternity patients, 3% of newborns and 39% of small and sick newborns are being cared for in an environment where nursing knowledge is very low (score <0.6).

**Relevance to clinical practice:**

Focus on periodic training, ensuring retention of knowledge and skills among health workers in low‐case load setting, and bridging the know‐do gap may help to improve the quality of care delivered to mothers and newborns in Kenya.

**What does this paper contribute to the wider global clinical community?**

- This study highlights the need to consider the competency of nurses as a dimension of quality of care, in addition to structural assessments and the process of medical care, during facility assessments.
- Gaps in nursing knowledge may be an important target area for quality improvement in high‐mortality settings if the Sustainable Development Goal 3 is to be achieved. More than a third of small and sick newborns accessing inpatient services in Nairobi City County were found to be doing so in a facility where nurses have poor knowledge (score <0.6) of how to provide them with quality care.
- Attention needs to be paid to improving nursing competency in managing hypertension in pregnancy and the care for small and sick newborns, including infant resuscitation and checking signs and symptoms of sick newborns.
- Regular training of nurses, particularly those within smaller facilities with low case‐loads of patients, may help improve competency of nurses providing maternal and newborn care.
- A questionnaire composed of vignettes and direct questions has been designed to evaluate nursing knowledge of maternal and neonatal care, including care for sick newborns. The questionnaire and standard operating procedures have been made available online for others wishing to adopt or adapt the tool for their setting.

## 1 INTRODUCTION

International efforts to improve maternal and child health have led to significant reductions in mortality over the last three decades (Victora et al., 2016). Further progress is required if the Sustainable Development Goal 3 of reducing maternal mortality to less than 70 per 100,000 live births and neonatal mortality to less than 12 per 1,000 live births by 2030 is to be achieved (United Nations). Neonatal mortality currently accounts for 45% of child deaths in Kenya, standing at 22 per 1000 live births (Kenya National Bureau of Statistics, 2015). Maternal mortality accounts for 14% of deaths among women aged 15–49, with a ratio of 362 per 100,000 live births (Kenya National Bureau of Statistics, 2015).

It is estimated that increased coverage and quality of essential interventions, many delivered through inpatient facility‐based care, could avert 71% of neonatal deaths, 33% of stillbirths, and 54% of maternal deaths by 2025 (Bhutta et al., 2014). The maximum effect on neonatal deaths would come from interventions delivered during labour and birth, such as emergency obstetric care and management of preterm labour, and care of small and ill newborns, such as resuscitation and management of neonatal infection (Bhutta et al., 2014).

In Nairobi City County, Kenya, 60%–70% of the population live in slums and income inequality is high (African Population and Health Research Center (APHRC), 2014). An estimated 88.7% of births take place within health facilities (50.1% in a public facility and 38.6% in a private facility), compared to 61.2% on a national level (Kenya National Bureau of Statistics, 2015). Yet, the neonatal mortality rate in Nairobi is considerably higher than elsewhere in Kenya (39 compared with 19–25 per 1,000 live births, respectively; Kenya National Bureau of Statistics, 2015). It is estimated that 18% of all newborns in the County will require inpatient services due to conditions such as infection, jaundice and prematurity in an NBU (Murphy et al., 2017). In 2015, Kenya's maternal mortality ratio remained high, standing at 510 per 100,000 live births (Keats et al., 2017). Maternal mortality has been estimated to be considerably higher in Nairobi slum populations compared with national estimates (Ziraba, Madise, Mills, Kyobutungi, & Ezeh, 2009).

## 2 BACKGROUND

Central to the delivery of essential healthcare services for maternal and newborn patients is adequacy of knowledge and skills of health workers, including nurses (Knight, Self, & Kennedy, 2013; van Lonkhuijzen, Dijkman, van Roosmalen, Zeeman, & Scherpbier, 2010; Renfrew et al., 2014). Assessments in low‐resourced settings have shown that, in many cases, structures are in place to support delivery of emergency obstetric and neonatal care (EmONC), but staff are unable to provide all the signal functions of EmONC (Kongnyuy, Hofman, Mlava, Mhango, & van den Broek, 2009; MEASURE Evaluation PIMA, 2016; Pattinson, Makin, Pillay, Van den Broek, & Moodley, 2015). Lack of knowledge and skills, together with health worker shortages, is likely to be a key reason why many essential interventions are not delivered or are delivered sub‐optimally to mothers and newborns in low‐resource settings.

Despite the delivery of quality care being dependent on the knowledge and skill of health worker, these are rarely assessed. Where assessments have been conducted, these have mostly been for maternal and immediate newborn care in public sector facilities, with less reported on care of small and sick newborns and from private facilities (Ameh et al., 2016; Ayiasi, Criel, Orach, Nabiwemba, & Kolsteren, 2014; Berhe, Tinsae, & Gebreegziabher, 2017; Traore et al., 2014). Approaches to assessing knowledge have increasingly emphasised the use of vignettes, which may provide more insight compared to traditional multiple choice questionnaires, while still remaining practical and standardisable (Patel et al., 2011; Peabody, Luck, Glassman, Dresselhaus, & Lee, 2000; Traore et al., 2014; Vesel et al., 2013).

Our study aimed to assess the knowledge of nurses, who are the main service providers for maternal, routine newborn, and small and sick newborn care, using vignettes and direct question approaches. The study further explores heterogeneity in knowledge between facilities of different types (sector, size and resource environment) and levels of training and experience of nurses. This study is part of a larger enquiry into the provision and quality of inpatient neonatal services in Nairobi City County, Kenya (Murphy et al., 2016).

## 3 METHODS

A cross‐sectional assessment of maternal and newborn care nursing knowledge in Nairobi City County, Kenya was conducted from July 2015–May 2016. Full details of the study protocol have been published elsewhere and all study tools made publicly available (Murphy et al., 2016; The Global Health Network). STROBE guidelines for observational research were followed in reporting this study (Supporting information).

### 3.1 Study setting

All health facilities from the public, private not‐for‐profit (mission) and private for‐profit (private) sectors providing inpatient neonatal care 24 hrs a day, 7 days a week (24/7) (hereafter referred to as INC facilities) in Nairobi City County, Kenya, were eligible to partake in the study; we explain how facilities were identified in a separate report (Murphy, Gathara, Abuya et al., 2018).

### 3.2 Knowledge questionnaire

A quantitative structured questionnaire was designed to assess the knowledge of nurses on the Kenyan guidelines for the care of maternity patients, routine newborn care on the delivery ward, and the care of small and sick newborns (or WHO guidelines where national guidelines were not available; Ministry of Health Republic of Kenya, 2013; Ministry of Medical Services & Ministry of Public Health and Sanitation Kenya, 2012). Maternity care questions focused on active management after birth, post‐partum haemorrhage, hypertension in pregnancy and maternal resuscitation. Routine newborn questions were on immediate care, care on the first day of live, breastfeeding and newborn resuscitation. Care of small and sick newborns included checking signs and symptoms on admission, management of common neonatal conditions and infant resuscitation. The questionnaire combined vignettes and direct questions and was developed by a research team of nurses, paediatricians and epidemiologists.

The questionnaire was designed in REDCap, an electronic data capture tool, with inbuilt checks and skips to ensure completeness, clarity and logical flow of questions (Harris et al., 2009). Three rounds of questionnaire piloting were conducted: first, with paediatrician researchers; second, with nurses experienced in working in both maternity units and NBUs; and third, the whole process of conducting knowledge testing was piloted in a county hospital outside of Nairobi (i.e., the facility was ineligible for the study). Some pilot interviews were conducted in pairs in order to develop common approaches to asking questions and consistency between the interviewers on how to interpret answers. Necessary changes to the tool and standard operating procedures (SOPs) were made at each step. The final tool and SOP are available in Supporting information.

The questionnaire was administered by an interviewer who, following strict SOPs, read each question out loud to the participant and recorded responses directly into the REDCap tool. Answer options were not provided to nurses; instead, nurses provided their own answers and the interviewer marked the multiple choice answers as appropriate. Two interviewers (AM and GN) conducted the survey, working together in each facility, interviewing in parallel when appropriate. Interviewers were research team members with extensive familiarity and training in the tool; they contributed to the design of the tool and writing the SOPs, and led piloting of the questionnaire.

### 3.3 Study population

All INC facilities in Nairobi City County were invited to partake in the study. Within participating facilities, nurses on duty in the maternity unit and NBU at the time of survey were eligible to partake in the nursing knowledge questionnaire. Demographic and training questions were asked to all participating nurses. Questions pertaining to care of maternity patients and routine newborn care were only asked to nurses on duty in the maternity unit. Questions pertaining to the management of small and sick newborns were only asked to nurses on duty in the NBU. Nurses on duty on both maternity and newborn units at the time of survey were asked all questions.

In units with three or fewer nurses on duty at the time of the survey, all nurses were invited to partake in the survey by the interviewers. In units with more than three nurses on duty, half of nurses were sampled. Nurses’ names in these units were numbered in alphabetical order; odd numbered nurses were selected and invited to partake. If a nurse declined to partake in the study, a replacement was taken from the list of remaining nurses starting from the top of the list and moving down until the desired sample was reached. Nurses offering care simultaneously on both the maternity unit and NBU at the time of survey were included in the sample for both units.

### 3.4 Knowledge scores

Knowledge scores were computed for each participant in a three‐step process. (1) Each question was scored 0 or 1 for incorrect or correct, respectively, as per national guidelines. Questions with multiple components (such as the vignettes) were scored 0 to 1 as a proportion of components correctly answered. (2) Scores from all questions relevant to a care topic (such as breastfeeding) were aggregated to produce a 0–1 score for the topic. (3) Combined scores of 0–1 for maternity care, routine newborn care, and for sick newborn care were created by aggregating scores for all relevant questions for each of these three types of care. In addition, we calculated mean scores for each facility by taking the mean knowledge scores of the nurses interviewed within a facility.

In order to explore the number of patients receiving care at different levels of knowledge, a facility's mean score was applied to the number of admissions to the facility between 1st July 2014–30th June 2015. Scores for maternity care and routine newborn care knowledge were applied to the number of maternal admissions at each facility (presumed one newborn per mother). Scores for care of sick newborns knowledge were applied to the number of neonatal admissions to the NBU at each facility. Admission numbers were obtained by review of maternal and newborn admission registers.

### 3.5 Structure and process scores

A structural assessment of infrastructure, equipment and drugs in the maternity and neonatal units was conducted and used to score facilities’ structural capacity to provide quality care between 0–100, as previously described (Murphy, Gathara, Abuya et al., 2018). Retrospective medical record review of neonatal admissions to the NBU was conducted to assess quality of process of care against pre‐specified indicators for quality of documentation, evidence of monitoring and correct treatment prescriptions (as per Kenyan national guidelines; Ministry of Health Republic of Kenya, 2013). These were used to create a process score 0–1 for each facility, as previously described (Murphy, Gathara, Mwachiro et al., 2018).

### 3.6 Data analysis

Data analysis was conducted using Stata version 13 (Stata Corporation, Texas, USA). Knowledge questionnaire results are presented as weighted means and proportions to account for the sampling of nurses, ensuring that each facility contributed to the results as a proportion of their contribution to the total number of nurses on duty at the time of survey.

Very large facilities were defined as those with >3000 maternity and >900 newborn, large as 1001–3000 maternity and 101–900 newborn, medium as 350–1000 maternity and 50–100 newborn and small as <350 and <50 newborn admissions between 1st June 2014–30th June 2015 for questions pertaining to maternity and routine newborn care and care of sick newborns, respectively.

The primary aim was to describe the overall knowledge of nurses and explore particular areas of weakness and strength. Secondary analyses were also conducted to explore how knowledge scores varied between facilities and nurses with different characteristics. To explore nursing characteristics, we fitted linear regression models adjust for clustering at the facility level and weighted using svy estimation commands in Stata. Facility characteristics were explored using linear regression; the unit of observation was the facility.

### 3.7 Ethics and permission

Ethical approval has been granted by the Kenya Medical Research Institute (KEMRI) Scientific and Ethics Review Unit (SSC protocol No. 2999). Written informed consent to conduct this study was obtained from the Medical Supervisor or equivalent authority in charge of each facility. Written informed consent was obtained from individual participants before administering the questionnaire.

## 4 RESULTS

Thirty‐three INC facilities were found to be eligible for the study. Two small private facilities declined to partake, and one participating facility provided paediatric services only. Hence, we report findings from 31 NBUs (four public, six mission and 21 private sector) and 30 maternity units (four public, six mission and 20 private sector).

Nurses were sampled in 15/30 maternity units and 11/31 NBUs, whereas all nurses on duty were interviewed in the remaining units. A total of 125 nurses were interviewed; 49 were on duty only on the maternity units, 42 were on duty only on the NBU, and 34 were on duty on both wards. Hence, 83 nurses (representing 65.4% of 127 eligible nurses) answered questions on maternity and routine newborn care and 76 nurses (representing 71.7% of 106 eligible nurses) answered questions on care of sick newborns. The number of nurses interviewed per hospital ranged from 1–12 (median = 3).

### 4.1 Nurse characteristics

**Figure 1:**
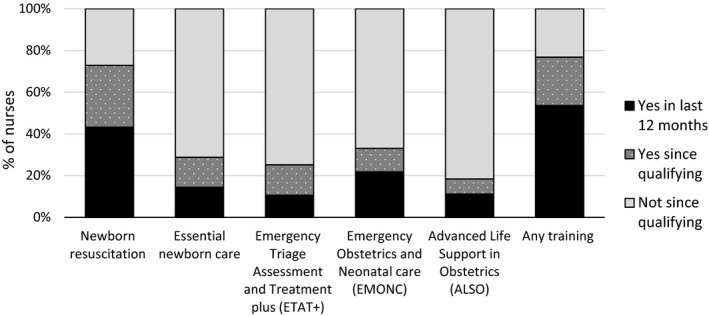
Training of nurses interviewed (*n* = 125). Any training: at least one of the five trainings presented

Most (62.4%) nurses interviewed were diploma nurses, 19.0% were specialist nurses (nine midwifery, two neonatal, one paediatric and two ICU/critical care nurses), 11.2% degree/BSN nurses, 4.8% enrolled/certificate nurses and 2.4% higher diploma nurses. All but nine nurses interviewed were women (7.2% men) and 88.8% were permanent staff in the facility in which they were interviewed. There was a wide range in the number of years of experience, from 0–35 years, averaging at 9.9 years. Half (53.7%) of nurses had completed at least one relevant training in the last 12 months (Figure 1).

### 4.2 Maternity care

The overall weighted mean score for knowledge of guidelines for management of maternity patients was 0.71 (95% CI: 0.65–0.77) though individual nurse scores varied widely (0.31–0.98).

Active management after birth (third stage of labour) was the best answered area of maternity care knowledge assessed (Table 1); the only component that was not well answered was that uterine massage should be done every 15 min for 2 hrs post‐delivery of the placenta, mentioned by only 8.1% of nurses (Appendix Figure S1).

**Table 1:** Overall weighted mean test scores for the nursing knowledge questionnaire

	Topic	All	Sector		
			Public	Mission	Private
Maternity care (*n* = 83)	Active management after birth	0.84 (0.80–0.87)	0.86 (0.77–0.94)	0.82 (0.75–0.89)	0.83 (0.80–0.87)
	Post‐partum haemorrhage	0.75 (0.70–0.81)	0.74 (0.64–0.84)	0.73 (0.66–0.79)	0.78 (0.70–0.85)
	Hypertension in pregnancy	0.58 (0.48–0.68)	0.49 (0.32–0.66)	0.58 (0.49–0.67)	0.62 (0.46–0.78)
	Maternal resuscitation	0.67 (0.57–0.77)	0.61 (0.28–0.93)	0.71 (0.61–0.80)	0.68 (0.61–0.75)
Routine newborn care (*n* = 83)	Immediate care	0.82 (0.78–0.86)	0.88 (0.83–0.93)	0.80 (0.72–0.88)	0.80 (0.74–0.86)
	Care on first day	0.72 (0.68–0.77)	0.76 (0.70–0.83)	0.73 (0.61–0.84)	0.70 (0.66–0.75)
	Breastfeeding	0.75 (0.70–0.80)	0.79 (0.65–0.92)	0.71 (0.62–0.80)	0.76 (0.71–0.81)
	Newborn resuscitation	0.66 (0.58–0.73)	0.68 (0.44–0.91)	0.63 (0.49–0.78)	0.66 (0.58–0.73)
Sick newborns (*n* = 76)	Signs and symptoms	0.58 (0.52–0.63)	0.67 (0.49–0.84)	0.51 (0.37–0.65)	0.57 (0.51–0.63)
	Management	0.80 (0.77–0.83)	0.88 (0.84–0.92)	0.76 (0.72–0.80)	0.80 (0.76–0.83)
	Infant resuscitation	0.55 (0.49–0.62)	0.61 (0.44–0.78)	0.51 (0.38–0.64)	0.55 (0.46–0.64)

A test score of 0 = 0% correct, a score of 1.0 = 100% correct. Results are presented as weighted mean (95% CI).

The weakest topic area of knowledge was management of hypertension in pregnancy (Table 1). Although almost all nurses (92%) knew that magnesium sulphate is the recommended drug for management of eclamptic convulsions, fewer (31%) nurses knew the correct loading and maintenance doses. When asked what should be monitored while administering magnesium sulphate for eclampsia to check for toxicity, only 33% mentioned all three checks for deep tendon reflexes, urine output and respiratory rate and 81% mentioned at least one.

The overall knowledge score for maternal resuscitation was 0.67 (95% CI: 0.57–0.77). Only 43% of nurses answered correctly when asked to list the four priority things that should first be done if a pregnant women, who is severely ill, collapses. When asked to state the recommended target breaths and cardiac compression rate per minute for cardiopulmonary resuscitation, only 2% of nurses provided a fully correct answer, with an additional 32% providing a partially correct answer.

### 4.3 Routine newborn care in the maternity unit

The overall weighted mean score for routine newborn care was 0.74 (95% CI: 0.69–0.78). As for maternity care, the crude range of results among nurses for routine newborn care was wide (0.37–0.98).

**Figure 2:**
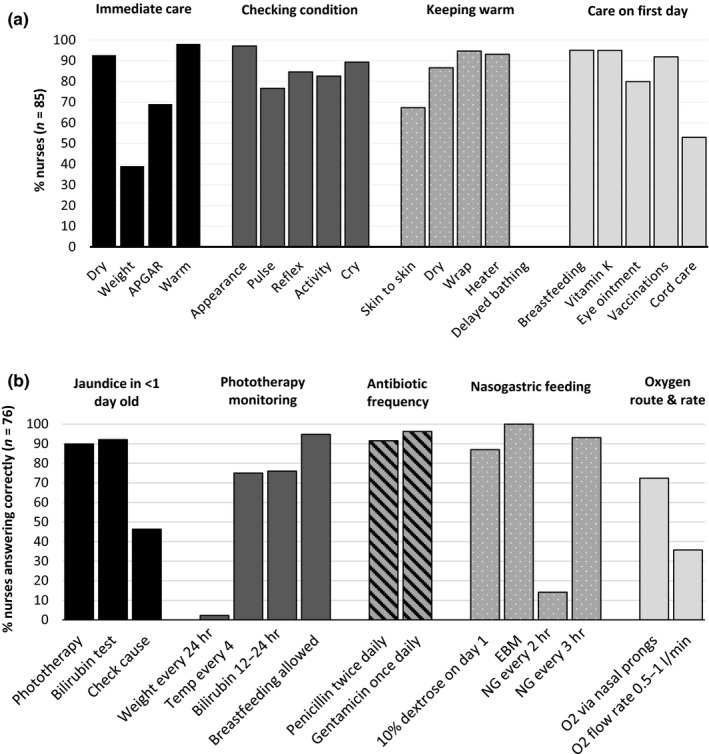
Questionnaire responses from nurses for (a) routine newborn care (*n* = 83) and (b) management of sick newborns (*n* = 76). temp: temperature; EBM: expressed breastmilk; NG: nasal gastric tube feeding; O_2_: oxygen treatment. Note: both NG every 2 hrs and NG every 3 hrs are correct

Nurses scored well when asked about the immediate care that should be given in the first few minutes after a normal delivery (Table 1). The only expected answer that was often missed was the need to weigh the baby; mentioned by only 39% of nurses (Figure 2a). With regard to the care that should be given to a well‐baby on the first day of life, good responses were given for breastfeeding, vitamin K administration, tetracycline eye ointment and BCG and oral polio vaccines (Figure 2a). However, only half (52.9%) of nurses mentioned that the umbilical cord needed to be cleaned.

All nurses answered that the most appropriate time to initiate breastfeeding is within the first hour/immediately after birth and that breastfeeding should ideally continue exclusively for 6 months. Almost all (99%) of nurses correctly answered that if a newborn is unable to breastfeed, but the mother is producing milk, then expressed breastmilk should be given. The overall score for this topic (Table 1) was reduced, however, by limited responses to the question on what to do if a mother is not producing enough breastmilk during the first few days, only 14.5% mentioned all five possible correct answers.

When asked direct questions, 98% of nurses knew that the recommended eye ointment is tetracycline; 88% and 86% mentioned oral polio vaccine and BCG, respectively, as the vaccines to be given to newborns; 77% of nurses correctly defined low birthweight as <2.5 kg; 73% and 72% knew that a PCR HIV test and a rapid (antibody) HIV test should be used for a newborn when mother is HIV positive and when the exposure status is unknown, respectively; and 67% of nurses knew that cord clamping should be delayed (Appendix Figure S2). Only 29% of nurses knew the correct dose of vitamin K and 14% that chlorhexidine is the recommended cord cleaning solution.

Nurse knowledge on newborn resuscitation was poor (Table 1). Most nurses provided at least partially correct answers for each step, with more than half of nurses answering fully correctly for the steps of checking the airways and checking breathing (Appendix Figure S3). The most commonly missed details were as follows: in step 1, only 33% of nurses mentioned that the newborn should be wrapped in a new/warm cloth/towel with the chest exposed; and in step 2, only 22% of nurses mentioned that the newborn should be re‐assessed after 1 min of ventilation.

### 4.4 Severely ill newborns in the newborn unit

The overall weighted mean score for the care of sick newborns was 0.62 (95% CI: 0.57–0.67). The range of knowledge scores for nurses was wide: 0.28–0.90.

The weakest areas answered were on checking signs and symptoms and infant resuscitation (Table 1). When asked what signs and symptoms should be checked for a sick newborn of 8 days old upon admission, the mean number of signs and symptoms mentioned was 6.9 of the 12 recommended by national guidelines (Ministry of Health Republic of Kenya, 2013). Most nurses provided at least partially correct answers for each step of infant resuscitation, with the exception of knowing the heart rate at which to stop resuscitating, which was correctly answered by only 37.2% of nurses (Appendix Figure S4).

Questions pertaining to the management of sick newborns were better answered (Table 1). Most nurses answered that phototherapy and bilirubin testing should be done for jaundiced babies <1 day old, but few mentioned that the cause of the jaundice should be checked (Figure 2b). Almost all nurses agreed that breastfeeding should be allowed during phototherapy. Three quarters knew that temperature and bilirubin should be monitored during phototherapy and the frequency of monitoring, but almost none (2.3%) mentioned that weight should also be monitored every 24 hrs.

Almost all nurses knew the frequency with which antibiotics and nasogastric feeding should be administered (Figure 2b). Knowledge of the guidelines on oxygen administration was less good, with only 36% of nurses knowing the correct flow rate, though 72% correctly answering that nasal prongs should be used. When asked what anti‐convulsant should be first used for a baby that is not on treatment having convulsions, 43% said diazepam (harmful for neonates), 67% said phenobarbitone and 9% said phenytoin (multiple answers were allowed).

### 4.5 Knowledge scores and nurse characteristics

Nurses with more years of experience and those who had received training additional training since qualifying tended have higher knowledge scores (Table 2). No statistically significant difference in knowledge scores was found between nurses had shared responsibilities between the maternity and newborn units and those who were on duty in only one unit (Table 2). In particular, ETAT+ and newborn resuscitation training were associated with higher sick newborn care and routine newborn care scores, respectively (*p*‐values 0.034 and 0.0006, respectively) (Appendix Table S1). Furthermore, nurses who had received training in newborn resuscitation training since qualifying performed better on newborn and infant resuscitation vignettes (*p*‐values 0.007 and 0.071, respectively).

**Table 2:** Weighted mean (95% CI) nurse knowledge scores by nurse characteristics

	Maternity care score	Routine newborn care score	Sick newborn care score
Dedicated nursing staff			
Yes (*n* = 49/42)	0.73 (0.64–0.81)	0.75 (0.69–0.81)	0.65 (0.58–0.71)
No (*n* = 34/34)	0.68 (0.61–0.74)	0.71 (0.65–0.77)	0.58 (0.51–0.65)
*p‐*Value	0.356	0.330	0.123
Years of experience			
0–2 (*n* = 15/17)	0.59 (0.52–0.66)	0.58 (0.52–0.65)	0.48 (0.42–0.54)
2–4 (*n* = 18/15)	0.71 (0.64–0.79)	0.77 (0.69–0.84)	0.59 (0.52–0.67)
4–10 (*n* = 23/17)	0.72 (0.63–0.80)	0.76 (0.69–0.83)	0.66 (0.60–0.73)
>10 (*n* = 27/27)	0.75 (0.67–0.84)	0.77 (0.72–0.82)	0.67 (0.60–0.74)
*p‐*Value^a^	0.136	**0.024**	**<0.001**
Any training since qualifying			
Yes (*n* = 58/59)	0.75 (0.69–0.80)	0.77 (0.73–0.82)	0.64 (0.59–0.69)
Yes in last 12 months (45/36)	0.75 (0.68–0.82)	0.77 (0.73–0.81)	0.65 (0.59–0.71)
No (*n* = 25/17)	0.61 (0.52–0.71)	0.65 (0.58–0.72)	0.52 (0.43–0.62)
*p‐*Value^b^	**<0.001**	**0.0034**	**0.022**

*n *= maternity care and routine newborn care scores/sick newborn care score.

A test score of 0 = 0% correct, a score of 1.0 = 100% correct. Results are presented as weighted mean (95% CI).

*p*‐Values were determined by fitting linear regression models on the scores (dependent variable) and the different nursing characteristics (independent variables) while adjusting for weighting and survey design. *p*‐values <0.05 are indicated in bold.

Dedicated nursing staff: nurses who were on duty only in the maternity unit or the newborn unit, not both, at the time of survey. Any training: training having been received in at least one of the five trainings: newborn resuscitation, essential newborn care, emergency triage assessment and treatment plus (ETAT+), emergency obstetrics and neonatal care (EMONC), or advanced life support in obstetrics (ALSO).

a *p*‐Value for log(years of experience) as a continuous variable.

b *p*‐Value compares those with and those without any training since qualifying.

### 4.6 Knowledge scores and facility characteristics

No statistically significant differences were found between average facility knowledge scores and facility sector or size, with the exception of larger facilities performing better in knowledge of sick newborn care (Table 3). Facilities with better structural capacity and better process of care scores also scored higher for knowledge of maternal and sick newborn care (Table 3).

**Table 3:** Mean (95% CI) facility knowledge scores by facility characteristics

	Maternity care score	Routine newborn care score	Sick newborn care score
Sector			
Public (*n* = 4)	0.72 (0.49–0.94)	0.77 (0.55–1.00)	0.73 (0.54–0.92)
Mission (*n* = 6)	0.70 (0.59–0.81)	0.71 (0.57–0.84)	0.59 (0.44–0.75)
Private (*n* = 20)	0.68 (0.62–0.74)	0.70 (0.64–0.76)	0.57 (0.50–0.63)
*p‐*Value public vs mission	0.834	0.415	0.140
*p‐*Value public vs private	0.630	0.325	**0.043**
Facility size			
Small (*n* = 9/16)	0.61 (0.50–0.71)	0.65 (0.56–0.74)	0.57 (0.49–0.64)
Medium (*n* = 9/3)	0.72 (0.66–0.78)	0.76 (0.68–0.83)	0.55 (0.33–0.78)
Large (*n* = 6/8)	0.72 (0.62–0.83)	0.71 (0.60–0.82)	0.58 (0.50–0.66)
Very large (*n* = 6/4)	0.73 (0.64–0.83)	0.75 (0.65–0.86)	0.75 (0.62–0.88)
*p‐*Value^a^	0.053	0.170	**0.031**
Structural score			
High (*n* = 9/10)	0.77 (0.74–0.80)	0.77 (0.70–0.85)	0.65 (0.59–0.71)
Medium (*n* = 11)	0.69 (0.62–0.77)	0.73 (0.64–0.81)	0.64 (0.53–0.74)
Low (*n* = 10)	0.62 (0.52–0.71)	0.65 (0.57–0.72)	0.49 (0.41–0.57)
*p‐*Value^b^	**0.009**	0.074	**0.010**
Process score			
High (*n* = 4)			0.75 (0.61–0.89)
Medium (*n* = 7)			0.60 (0.52–0.68)
Low (*n* = 17)			0.55 (0.47–0.63)
*p‐*Value^b^			**0.014**

*n *= maternity care and routine newborn care scores/sick newborn care score.

A test score of 0 = 0% correct, a score of 1.0 = 100% correct.

Facility size: number of admissions 1st June 2014–30th June 2015 of maternity patient (small <350, medium 350–1000, large 1001–3000, very large >3000) for maternity care and routine newborn care scores and the number of neonatal patient (small <50, medium 50–100, large 101–900, very large >900) admissions for sick newborn care score.

*p*‐Values are for unadjusted linear regression, the unit of observation is the facility. *p*‐values <0.05 are indicated in bold.

a *p*‐Values are for log(admission numbers) as a continuous variable.

b *p*‐Values are for scores as continuous variables.

### 4.7 Care for maternal and neonatal patients

**Figure 3:**
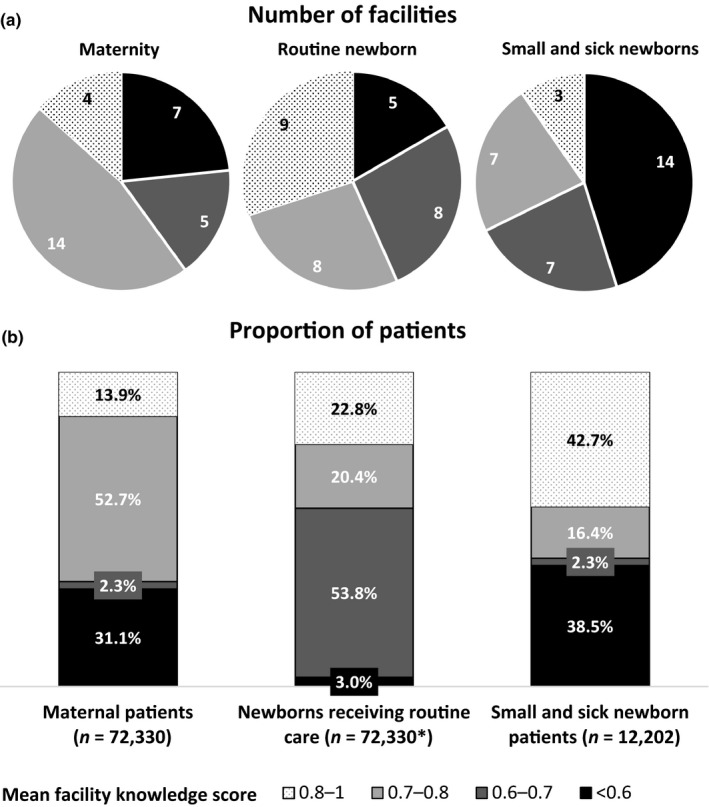
Number of facilities (a) and proportion of patients receiving care at those facilities (b) by level of nursing knowledge. *n*: total number of patients obtained from review of patient admission registers. *Presumed one newborn per maternal patient [Colour figure can be viewed at wileyonlinelibrary.com]

Mean knowledge scores were high (0.8–1) in four, nine and three facilities but very poor (<0.6) in seven, five and 14 facilities for knowledge of maternity care, routine newborn care and care for sick newborns, respectively (Figure 3a). When facility scores were applied to the number of admissions at each facility (Figure 3b), the largest proportion (52.7%, *n* = 72,330) of maternity admissions to study facilities were cared for in a facility where nurses scored a mean of 0.7–0.8 in their knowledge of maternity care. The largest proportion (53.8%, *n* = 72,330) of newborns were cared for in a facility where nurses scored a mean of 0.6–0.7 in their knowledge of routine newborn care. Most inpatient small and sick newborns were admitted to facilities with either very high (score 0.8–1, 42.7% of patients, *n* = 12,202) or very poor (score <0.6, 38.5% of patients, *n* = 12,202) nursing knowledge scores on sick newborn care.

## 5 DISCUSSION

Complications during childbirth and failure to deliver adequate maternal and immediate newborn care contribute significantly to maternal and neonatal mortality and morbidity (Bhutta, Lassi, Blanc, & Donnay, 2010; Lassi, Majeed, Rashid, Yakoob, & Bhutta, 2013; Lawn et al., 2011). When complications occur, it is vital that key EmONC interventions are delivered and newborns have access to quality inpatient services delivered by competent health workers in an adequately resourced health facility (Bhutta et al., 2014; Kerber et al., 2007; Moxon et al., 2015). Overall, we found that nurses interviewed demonstrated a good to fair level of knowledge of guidelines for management of maternity patients and routine newborn care, but knowledge of guidelines for small and sick newborns care was poorer and concerning gaps in knowledge were identified. Given the central role of nurses in providing day‐to‐day care for maternal and newborn patients and the frequent absence of specialist medical personnel in our setting, it is crucial that nurses have adequate knowledge and competence to deliver quality care.

Several studies from other low‐resourced settings have also found deficits in health worker knowledge for maternal and newborn care (Ayiasi et al., 2014; Berhe et al., 2017; Mirkuzie, Sisay, Reta, & Bedane, 2014; Traore et al., 2014). The low knowledge of resuscitation for maternal, newborn or infant patients found in our study is particularly concerning. The ability to resuscitate a mother or newborn is of vital importance to reducing maternal and newborn mortality (Bhutta & Black, 2013; World Health Organization, 2016). A previous study from Kenya also found that knowledge of neonatal resuscitation was poor with only 35% of health workers scoring above 85% on stating the steps of resuscitation (Murila, Obimbo, & Musoke, 2012).

Nurses working in facilities with few admissions and facilities that were poorly equipped with essential maternal and newborn equipment and supplies tended to perform less well in our assessment. Maintaining competencies is challenging in facilities with low caseloads and, therefore, limited opportunity to practice skills. Lack of resources may also lead to guidelines not being implemented. For example, we found that only 14% of nurses said that chlorohexidine is the recommended cleaning solution for cord care; this is likely due to the lack of availability of chlorohexidine in many facilities in Nairobi. Other studies have also shown that the characteristics of the work environment can impact the competency of healthcare workers (Berhe et al., 2017; Kim et al., 2013; Traore et al., 2014). In Ethiopia, health facilities having adequate materials for newborn care were almost five times more likely to practice good newborn care compared to health facilities with shortages (Berhe et al., 2017). In Afghanistan, the facility setting was found to impact providers’ knowledge (with larger facilities performing better) but not their skills in neonatal resuscitation (Kim et al., 2013). The authors conclude that this may reflect differences in learning environments with larger facilities having specialised physicians and medical residents on staff and actively promoting continuing education (Kim et al., 2013).

Indeed, periodic training for health workers may help to improve knowledge and skills retention, especially in low case‐load facilities (Ameh et al., 2016; Ariff et al., 2010; McClure et al., 2007). In this study, we found that nurses who had received additional training since qualifying performed considerably better than those who had not. A previous study in Kenya found that more than 70% of health workers considered their knowledge about neonatal resuscitation inadequate and blamed it on inadequate medical training programmes (Murila et al., 2012). Yet another study from Kenya found that implementation of an EmONC training course increased knowledge and skill score from 61.3 to 82.8 among 1,636 health workers (Ameh et al., 2016).

Knowledge assessments, such as the one we have conducted in this study, may be a useful complement to Service Availability and Readiness Assessment surveys for identifying where and on what topics training is most needed (World Health Organization, 2015). Where periodic training cannot be maintained, care might be better centralised in larger better resourced facilities. If such an approach is to be considered then adequate referral systems will need to be in place and current overcrowding and staff shortages in larger Nairobi hospitals would need to be addressed to accommodate a larger volume of patients (Murphy, Gathara, Abuya et al., 2018).

Our study has both strengths and limitations. Our sampling approach was not proportional to the nurses on duty at each facility; small and private facilities were overrepresented. We addressed this limitation by applying proportional weighting to the analysis. The sample size restricted our ability to perform extensive modelling to explore associations between knowledge and characteristics of nurses and facilities. However, our aim was instead to provide a description of key areas of strength and weakness and descriptively explore heterogeneity of knowledge. The questionnaire we used cannot be considered a comprehensive assessment of staff competency in maternal and newborn health, and we were unable to validate the tool. However, it likely provides a good proxy of knowledge, is practical and quick to administer with few resources, takes local guidelines into account, and incorporates clinical vignettes, which have been advocated as a valid and reliable method to measure the competence of healthcare workers (Peabody et al., 2000). Interviewers were highly trained to interpret responses given by nurses to determine whether or not the answer was correct. A balance was carefully sought to ensure that nurses were not prompted to give the correct answer by the interviewer, yet also ensuring that incorrect answers were not recorded due to miscommunication between the interviewer and the nurse (e.g., interviewers were permitted, as per the SOPs, to ask the interviewee to explain what they mean by the answer they have given). Nonetheless, it is possible that errors or biases may have occurred due to misunderstandings between the interviewer and interviewee.

## 6 CONCLUSION

More than a third of small and sick newborns accessing inpatient services in Nairobi City County are doing so in a facility where nurses have poor knowledge (score < 0.6) of how to provide them with quality care. Previous reports have suggested that 44% of Nairobi's newborns are not accessing any INC facility when needed, and a further 30.4% are receiving inadequate services (Murphy et al., 2017; Murphy, Gathara, Mwachiro et al., 2018). This study highlights the need to consider the competency of nurses as a dimension of quality of care in addition to structural assessments and the process of medical care.

## 7 RELEVANCE TO CLINICAL PRACTICE

Quality of care provided during labour and childbirth is a determinant of the health outcomes for both mothers and their babies. Where complications do occur, it is vital that facilities are adequately prepared to manage inpatient newborn patients or appropriately refer. The competency and ability of healthcare workers to identify problems and act in accordance with standardised evidence‐based guidelines is key if progress is to be made in further reducing maternal and newborn mortality. This study reports strength and weakness in knowledge among nurses practicing in a setting with high neonatal mortality; highlighting topics that require improved focus during training.

## CONFLICT OF INTEREST

None declared.

## AUTHORS’ CONTRIBUTIONS

GM designed the study, with extensive input from all authors; GM and DG supervised data collection; JM and NA coordinated data collection; AM and GN collected the study data; GM analysed the data and wrote the manuscript, with support from ME and DG; All authors reviewed and provided input into the final version of the manuscript.

## AVAILABILITY OF DATA AND MATERIAL

The source data are owned by the Kenyan Ministry of Health, County Governments and individual private and mission hospitals and as the data might be used to de‐identify hospitals the study authors are not permitted to share the source data directly. Users who wish to reuse the source data are able to make a request initially through the KEMRI‐Wellcome Trust Research Programme data governance committee. This committee will supply contact information for the KEMRI Scientific and Ethical Review unit, Nairobi County, and individual hospitals as appropriate. The KEMRI‐Wellcome Trust Research Programme data governance committee can be contacted on: dgc@kemri-wellcome.org.
