# Supplementary material for: Nursing knowledge of essential maternal and newborn care in a high‐mortality urban African setting: A cross‐sectional study
Source: J Clin Nurs. 2018 Nov 26;28(5-6):882–93. doi: 10.1111/jocn.14695 (PMC6472564; doi:10.1111/jocn.14695)
Supplement: Supplementary file 1 [file JOCN-28-882-s001.docx]

**Appendix**

**Appendix table S1:** Training and weighted mean (95% CI) knowledge scores

|  | **Maternity care score** | **Routine newborn care score** | **Sick newborn care score** |
| --- | --- | --- | --- |
| **ALSO training** |  |  |  |
| In last 12 months (n=12) | 0.77 (0.70-0.83) |  |  |
| Since qualifying (n=7) | 0.82 (0.75-0.89) |  |  |
| Not since qualifying (n=64) | 0.68 (0.62-0.75) |  |  |
| *p-value* | ***0.004*** |  |  |
| **EMONC training** |  |  |  |
| In last 12 months (n=20) | 0.72 (0.62-0.81) | 0.76 (0.71-0.82) |  |
| Since qualifying (n=13) | 0.78 (0.74-0.82) | 0.79 (0.71-0.88) |  |
| Not since qualifying (n=49) | 0.69 (0.61-0.77) | 0.72 (0.66-0.77) |  |
| *p-value* | *0.218* | *0.085* |  |
| **Essential newborn care training** |  |  |  |
| In last 12 months (11/12) |  | 0.80 (0.72-0.88) | 0.69 (0.61-0.78) |
| Since qualifying (9/10) |  | 0.69 (0.62-0.76) | 0.61 (0.54-0.68) |
| Not since qualifying (63/54) |  | 0.73 (0.68-0.79) | 0.61 (0.55-0.67) |
| *p-value* |  | *0.754* | *0.264* |
| **ETAT+** **training** |  |  |  |
| In last 12 months (n=8/10) |  | 0.83 (0.76-0.91) | 0.72 (0.65-0.79) |
| Since qualifying (n=6/15) |  | 0.75 (0.63-0.88) | 0.64 (0.57-0.72) |
| Not since qualifying (n=68/50) |  | 0.73 (0.68-0.77) | 0.59 (0.53-0.65) |
| *p-value* |  | *0.052* | ***0.034*** |
| **Newborn resuscitation** **training** |  |  |  |
| In last 12 months (n=35/31) |  | 0.78 (0.73-0.82) | 0.65 (0.59-0.71) |
| Since qualifying (n=18/26) |  | 0.78 (0.72-0.84) | 0.63 (0.56-0.70) |
| Not since qualifying (n=30/19) |  | 0.67 (0.60-0.73) | 0.54 (0.45-0.64) |
| *p-value* |  | ***0.006*** | *0.076* |
|  |  | **Newborn resuscitation** | **Infant resuscitation** |
| **Newborn resuscitation** **training** |  |  |  |
| In last 12 months (n=35/31) |  | 0.72 (0.63-0.81) | 0.60 (0.52-0.69) |
| Since qualifying (n=18/26) |  | 0.72 (0.62-0.82) | 0.56 (0.48-0.64) |
| Not since qualifying (n=30/19) |  | 0.54 (0.43-0.65) | 0.43 (0.28-0.58) |
| *p-value* |  | ***0.007*** | *0.071* |

*n= routine newborn care scores / sick newborn care score. Results are presented as weighted mean (95% CI).*

*P-values were determined by fitting linear regression models on the scores (dependent variable), adjusted for weighting and survey design using the svy command in Stata, comparing those who had with those who had not received any training since qualifying (independent variable).*

**Figure S1:** Active management of third stage labour

**Figure S2:** Direct questions about guidelines for routine newborn care (n=83)

*Abbreviations: Polymerase chain reaction (PCR), Bacillus Calmette-Guérin vaccine (BCG), oral polio vaccine (OPV), tetracycline (TEO), vitamin K (VitK), low birth weight (LBW)*

**Figure S3:** Proportion of each step answered when describing newborn resuscitation

**Figure S4:** Proportion of each step answered when describing infant resuscitation
